# No neuropathological evidence for a direct topographical relation between microbleeds and cerebral amyloid angiopathy

**DOI:** 10.1186/s40478-015-0228-9

**Published:** 2015-08-14

**Authors:** Enikö Kövari, Andreas Charidimou, François R. Herrmann, Panteleimon Giannakopoulos, Constantin Bouras, Gabriel Gold

**Affiliations:** Department of Mental Health and Psychiatry, University Hospitals and University of Geneva, 2, chemin du Petit-Bel-Air, 1225 Geneva, Switzerland; Massachusetts General Hospital Stroke Research Center, Harvard Medical School, 175 Cambridge Street, Suite 300, Boston, MA 02114 USA; Department of Internal Medicine, Rehabilitation and Geriatrics, University Hospitals and University of Geneva, 3 chemin du Pont-Bochet, 1226 Thônex, Switzerland

## Abstract

**Introduction:**

Cerebral microbleeds correspond to blood breakdown products, including hemosiderin-containing macrophages around small vessels on histological examination. Superficial lobar cerebral microbleeds are increasingly recognized on MRI as a biomarker of cerebral amyloid angiopathy but the direct association between amyloid-laden vessels burden and cerebral microbleeds has yet to be validated neuropathologically. To address this issue, we examined the frequency of histopathologically-defined cerebral microbleeds in different brain regions and their relationship with cerebral amyloid angiopathy in a large autopsy population.

**Results:**

The frontal, parietal and occipital cortex as well as the adjacent white matter and basal ganglia of 113 consecutive autopsies were examined. Cerebral microbleedss were identified on haematoxylin-eosin-stained histological slides, cerebral amyloid angiopathy using anti-amyloid antibody. Cerebral microbleeds were present in 92.9 % of the cases and cerebral amyloid angiopathy in 44.3 % of them. Cerebral microbleeds were more frequent in parietal and frontal lobes followed by the occipital region and basal ganglia. In contrast, cerebral amyloid angiopathy was most frequent in the occipital lobe. There was no significant topographical association between cerebral amyloid angiopathy presence or severity and cerebral microbleeds in any brain region. In lobar areas, cerebral amyloid angiopathy was found in the cortex, predominantly affecting pial arteries and their superficial cortical branches, in contrast to microbleeds which were mainly in the white matter and occurred around deeper arteries and arterioles, including the subcortical segment of long penetrating branches of pial vessels.

**Conclusions:**

Our study does not support a direct relation between cerebral microbleeds and cerebral amyloid angiopathy burden at the neuropathological level, raising intriguing questions on the potential pathophysiological mechanisms of cerebral microbleeds in the context of cerebral amyloid angiopathy or other small vessel disease pathology.

## Introduction

Cerebral microbleeds (CMB), an MRI marker of small vessel disease in the brain, are increasingly recognized in healthy elderly individuals and patients with Alzheimer’s disease (AD) or cerebrovascular pathologies and vascular cognitive impairement. Radiologically, CMB are defined as small, rounded, hypointense lesions on blood-sensitive MRI sequences, inlcuding T2*-weighed gradient echo sequences and susceptibility-weighted imaging (SWI) [1, 2]. Limited neuropathological studies indicate that the majority of these radiologically-defined lesions correspond to haemosiderin containing macrophages around small vessels, mainly arterioles [3]. Despite the current hypothesis of CMBs being a primarily haemorrhagic marker of small vessel injury, the pathophysiological mechanisms and histopathological basis of CMBs remain poorly understood and highly debated [4]. In fact, recent neuropathological observations indicate hemosiderin within macrophages in the brain might not always be indicative of CMB but rather being associated with iron clearance [5]. In addition, careful pathologic analysis in a population-based autopsy study has demonstrated that a proportion of cerebral microbleeds are associated with microinfarcts [6]. Hence, multiple mechanisms seem likely to contribute to development of microbleeds [7].

Circumstantial evidence supports the hypothesis that hypertensive arteriopathy is mainly associated with deep CMB (i.e. in the basal ganglia, thalami etc.), whereas cerebral amyloid angiopathy (CAA) is notably related to superficial, lobar CMBs [3, 5, 8–15]. Since CAA pathology is not directly visible on neuroimaging, the *in vivo* clinical diagnosis of CAA is currently based on the recognition of multiple strictly lobar CMB on MRI, as a putative marker of the disease [9, 16–19]. This approach and more importantly the exact mechanism by which CAA gives rise to CMB has not been explored in neuropathological studies. Only a handful of cases have been examined for histopathological confirmation of MRI data [3, 12, 14, 20, 21]. Even fewer studies have examined the direct neuropathological correlation between CAA and lobar CMB [22].

## Materials and methods

The study received approval from the Ethics Committee of the University of Geneva. Brains were obtained by autopsies from the Department of Internal Medicine, Rehabilitation and Geriatrics, Division of Geriatrcs, and the Department of Readaptation and Palliative Medicine, University Hospitals, Geneva. We included 113 consecutive brain autopsies – 33 cases with dementia - performed between August 2012 to August 2014. For neuropathological analysis, tissues blocks were systematically taken in all cases from the hippocampus, inferior temporal cortex (Brodmann area 20), frontal cortex (Brodmann area 9), parietal cortex (Brodmann area 40), occipital cortex (Brodmann areas 17 and 18) and the basal ganglia at the level of the anterior commissure of the left cerebral hemisphere. Paraffin-embedded 14-μm-thick adjacent sections were stained with haematoxylin-eosin (HE), cresyl-violet (Nissl) and using anti-Aβ monoclonal antibodies (4G8; Signet Laboratories, Dedham, MA, USA; 1:2000) and anti-tau (AT8; Pierce Biotechnology, Rockford, IL, USA; 1:1000) consecutively. The severity of AD-related neuropathological changes was defined using the Braak stages for neurofibrillary tangles (NFTs) and the Thal phases for amyloid plaque deposition [23, 24] in the medial temporal lobe.

The presence or absence of CMB was noted on HE-stained histological sections. A case was considered positive for CMBs when haemosiderin-laden macrophages were present around at least one vessel, in one or more of the sampled regions. The presence or absence of CAA was determined on anti-Aβ-antibody-stained sections. In the basal ganglia only grey matter lesions but no lesions in the surrounding white matter or internal capsule were taken into consideration.

**Fig. 1:**
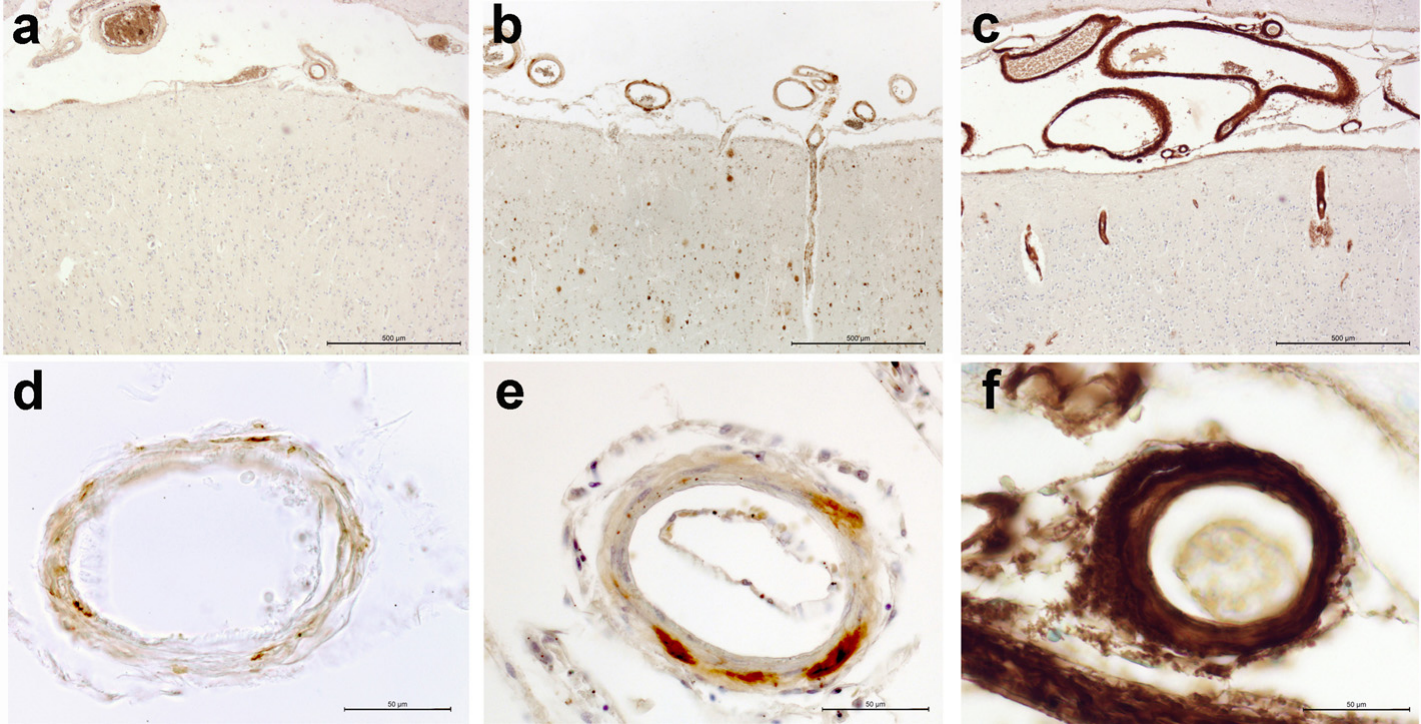
Grading of the severity of amyloid angiopathy. Grade 1: scattered amyloid deposition presenting as a thin rim of Aβ positive fibers around the smooth muscle cell of the media (**a** and **d**); Grade 2: more extensive presence of amyloid in the vessel’s wall with focal replacement of the muscular layer (**b** and **e**); Grade 3: complete destruction of the wall by the severe amyloid deposition, present in most pial and superficial vessels (**c** and **f**). The absence of amyloid, grade 0 not shown in the figure. (4G8 anti amyloid antibody (**a**-**f**). Scale bars:a,b and c: 500 μm; d, e and f: 50 μm)

On a four-level grading system CAA severity and burden was evaluated based on the existing systems of Vonsattel and al. [25] and Olichney et al. [26]. The average severity of amyloid deposition in the vessels wall and the extent of the lesions were taken in account as follows: grade 0 was noted in the absence of CAA. In grade 1 only a thin amyloid-positive rim was observed around smooth muscle cells of the otherwise normal media layer of scattered vessels (Fig. 1a, d). Grade 2 showed more extensive depositions and the medial layer of the vessels replaced by amyloid (Fig. 1b, e). Grade 3 was characterized by circumferential, severe destruction of the wall by amyloid deposition, with most vessels being affected (Fig. 1c, f). Separately, in each case the exact localisation (meningeal or meningo-cortical) as well as the presence and severity of capillary amyloid deposition [27] was noted as recently proposed by Love et al. [28]. The severity of capillary amyloid deposition was defined on ground of its extension.

### Statistical analysis

Group comparisons were performed using Fisher’s exact test for dichotomous variables, Student *t*-test for continuous variables or Wilcoxon–Mann–Whitney *u*-test for ordinal variables as appropriate. Multivariable logistic regression analyses were used to evaluate the association between the dependent variables (CMB or CAA) within each brain area and the independent variables (Braak NFT staging, Aβ deposition staging) with and without adjustment for age. Significance level was set at 0.05 and all tests of significance were two-tailed. All statistical analyses were carried out using Stata version 13.1 (Stata Corporation, College Station, TX, USA).

## Results

Our study sample consisted of 64 women and 49 men (mean age: 81.1 ± 10.8 years; age range: 51 – 99 years; Table 1). Macroscopic intracerebral haemorrhage was found only in two cases, both in subtentorial regions (brainstem and cerebellum). Lobar CMB were found in 92.9 % of the study population whereas CAA was present in 44.3 % of the cases. In the different diagnostic groups distinct prevalences of CAA was observed, the highest in the 25 cases with AD (Braak stage ≥ 4; 14 pure AD and 11 associated with vascular encephalopathy – Table 2). The prevalence of CMBs was highest in the parietal (84.1 %) and frontal lobes (76.9 %), followed by the occipital area (46.9 %). In contrast, CAA was more frequent in the occipital lobe (38.9 %), compared to frontal (30.9 %) and parietal areas (28.3 %). Parenchymal (cortical) CAA was most frequent in the occipital area, in 72.3 % of CAA cases (Table 3).

**Table 1:** Demographic data

	Number of cases	Mean age ± SD
Total (M/F)	113 (49/64)	81.1 ± 10.8
Non demented	80	79.1 ± 11.7
Dementia	33	85.7 ± 6.4
AD	14	84.8 ± 7.1
VaD	5	88.2 ± 3.6
Mixed D	11	86.2 ± 7.1
LBD	3	84.3 ± 6.1

*AD* Alzheimer’s disease, *VaD* vascular dementia, *Mixed D* mixed (AD and vascular) dementia, *LBD* Lewy body dementia

**Table 2:** Frequencies of CAA and CMB and mean CAA severities depending on neuropathological diagnosis

Neuropathologial diagnosis	No of cases	Prevalence of CAA (%)	Prevalence of CMB (%)	Mean severity of CAA
AD	14	71	78	1.5
Va	29	55	93	1.2
AD + Va	11	63	91	1.7
C	34	38	91	0.7
Others	25	16	100	0.5

*AD* Alzheimer’s disease, *Va* vascular encephalopathy, *AD + Va* mixed (AD-type and vascular encephalopathy), *C* no brain lesions, *Others* = any type of Lewy body pathology as Parkinson’s disease, Lewy body dementia – 8 cases, or multiple system atrophy (1 case), amyotrophic lateral sclerosis (2 cases) or brain metastases (14 cases)

**Table 3:** Distribution of CAA and the proportion of meningeal and meningocortical CAA in the different brain regions

Region (total number of CAA)	CAA (%) of the total population	mx_CAA (%)	mx_cx_CAA (%)
Frontal (35)	30.9	18 (51.4)	17 (48.6)
Parietal (32)	28.3	13 (40.6)	19 (59.4)
Occipital (44)	38.9	12 (27.3)	32 (72.3)

*Mx_CAA* CAA present only in meningeal vessels, *mx_cx_CAA* CAA present in both meningeal and cortical vessels

Basal ganglia CMBs were identified in 46.2 % of the cases. CAA in the basal ganglia was seen in 1 case only. With the exception of 3 cases, basal ganglia CMBs were accompagnied by lobar CMBs in at least one cortical area.

Capillary amyloid deposition was observed in 12 subjects (24 % of CAA positive cases and 10 % of the total population). Seven cases showed moderate-severe, 5 mild amyloid deposition. Capillary amyloid deposition (type 1) [27] or its severity did not influence the presence or absence of CMB (frontal lobe: *p* =0.209; parietal lobe: *p* =0.379; occipital lobe: *p* =0.736).

**Fig. 2:**
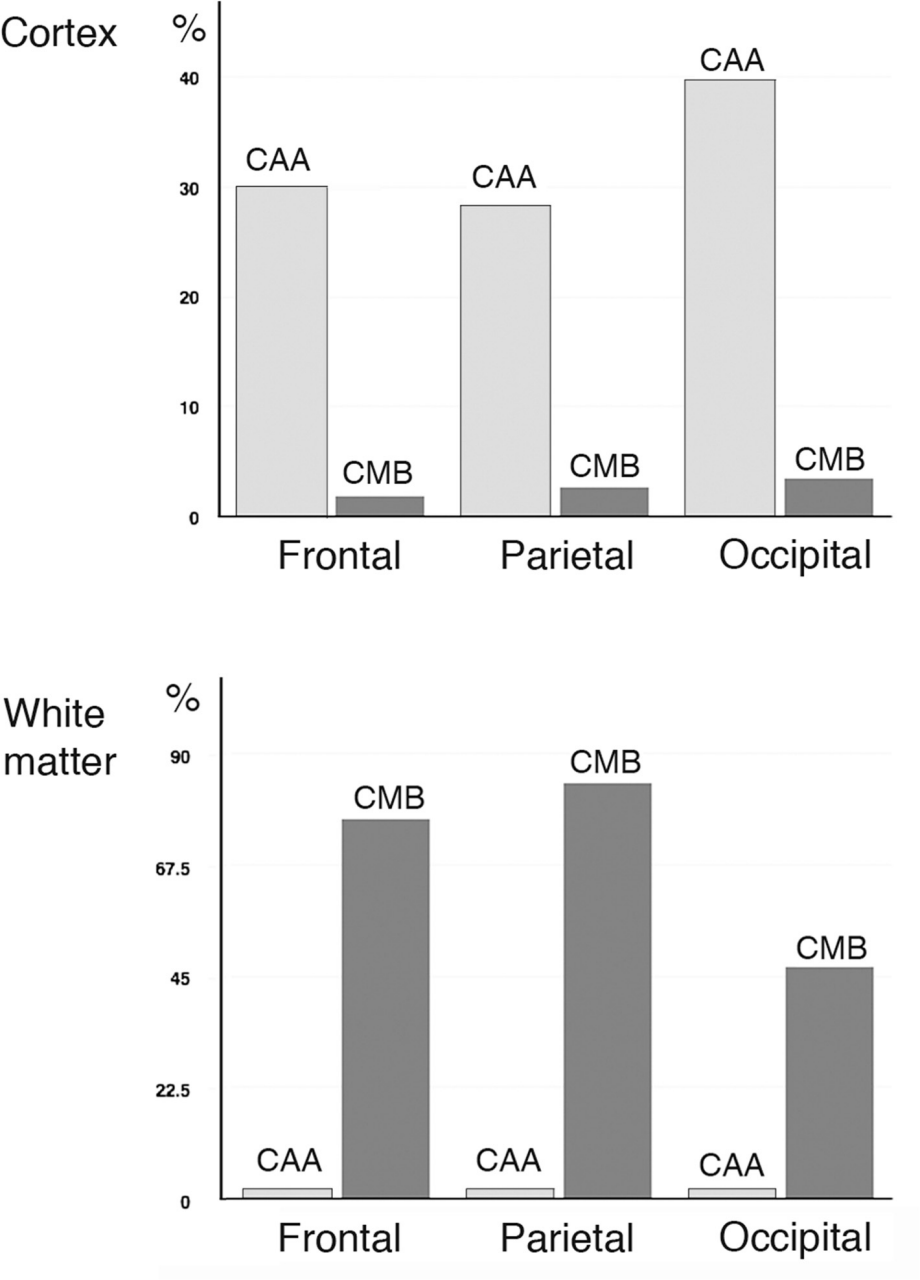
Percentage of cases with CMB and CAA. Distribution of CAA and CMB in the cortex and subcortical white matter. Note that CMB and CAA occur in different sectors of the brain (see text for details)

**Fig. 3:**
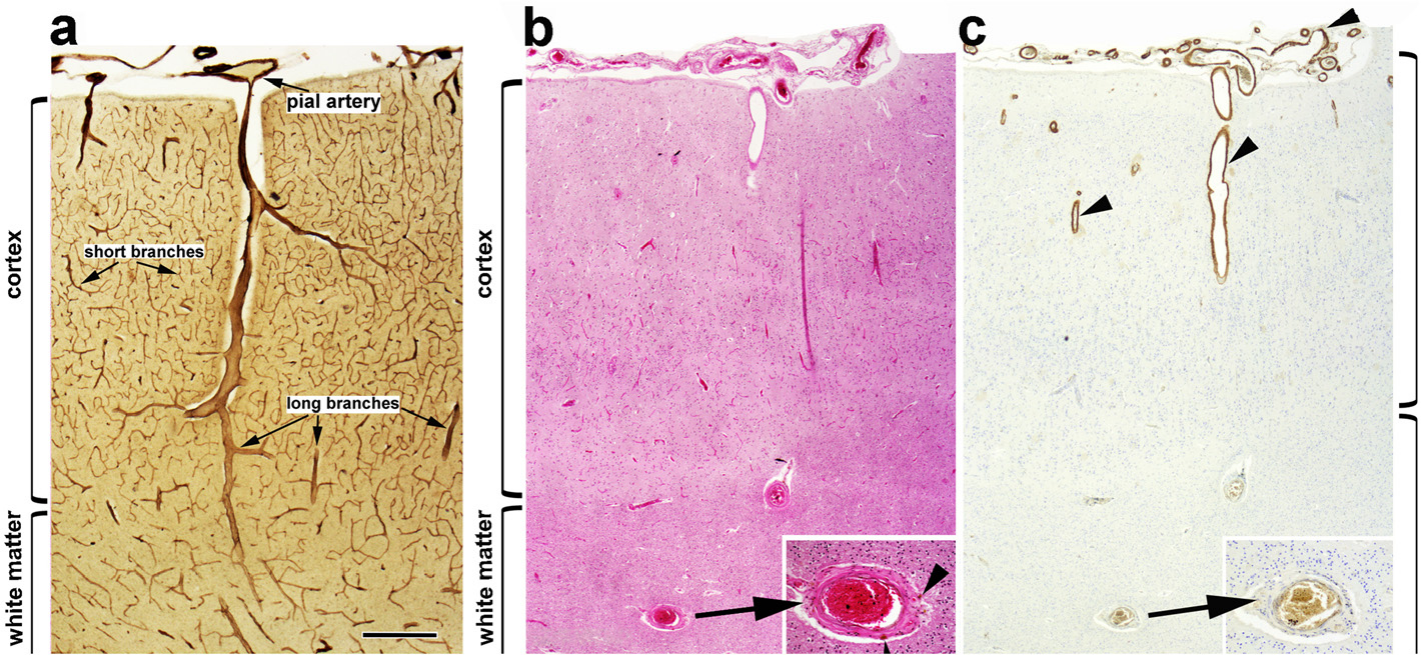
Topographical distribution of CMB and CAA. **a** Normal microvascular architecture of the cerebral cortex and underlying white matter. **b** Hemosiderin-laden macrophages (arrowheads) around small arteries of the white matter. **c** Amyloid angiopathy (arrowheads) in the pial and superficial cortical branches. **a**: modified Gallyas silver-impregnation; **b**: haematoxylin-eosin staining; **c**: immunohistochemistry with anti-amyloid antibody 4G8. Scale bar on A: 500 μm)

There was no significant association between the presence of CAA and lobar CMB either overall (Fisher’s exact *p* =0.463) or separately in each brain region examined. CAA, was associated with age (*p* < 0.001) but CMB were not. CAA in the white matter was rare (5 cases, 4.4 %) and only 8 cases (7 %) exhibited intracortical CMBs, indicating that these lesions occur in different compartments of the brain (Fig. 2). On close topographical analysis, CAA and CMB involved different types or different segments of vessels. CAA was mainly present in the pial arteries and their superficial short perforating branches, and less often in the superficial (intra-cortical) segments of long branches, e.g. in the surface of the brain or in the superficial cortical layers. In contrast, lobar microbleeds were detected in the adjacent white matter, often at the cortical-white matter junction (i.e. juxtacortical at the level of and around the long branches of pial vessels and the medullary arteries) or around deeper arteries and arterioles (Fig. 3).

We explored whether CMB presence in the underlying juxtacortical white matter was related to leptomeningeal/cortical CAA. We found no association in any of the studied regions for either the presence (Fisher’s exact test *p* = 0.628; 0.776; and 0.701) or severity (Mann–Whitney *p* =0.326; 0.656; 0.628) of leptomeningeal/cortical CAA in frontal, parietal and occipital lobes, respectively.

## Discussion

Our report is the first systematic neuropathological study in a large autopsy series directly examining the relationship between CMB and CAA, including their anatomical distribution. According to our primary findings there is no association between the overall presence and burden of CAA and histopathologically-defined CMB presence, nor concerning their topography within brains. These results raise interesting questions about the precise mechanisms underlying the development of most microbleeds.

We report a high frequency of CAA which is in line with earlier results from our group [29] and other published data from consecutive autopsy cases that used immunohistochemical detection methods [30, 31]. CMBs were more frequently detected in the current cohort compared to other autopsy based studies. This may be related in part to strict adherence to the neuropathological definition and, close attention to the presence of CMBs since their identification was a main aim of the study. Fisher et al. reported a CMB prevalence of 66 % in 33 autopsy cases [32]. In a large population-based autopsy study of older people with intracerebral hemorrhage (*n* = 300), Tanskanen et al. investigated the presence and extent of CMBs [6] and found them in 67.3 % of the cases. This confirms that neuropathological-defined CMB on histologic examination are much more common compared to rediologically-defined CMBs.

Our results do not confirm a direct neuropathological relationship between lobar CMB and CAA. This is consistent with a recent study of cerebellar microbleeds by de Reuck et al. who reported that CMB were related to atherosclerosis but not to CAA [33]. This contrasts with the bulk of the neuroimaging-based literature [15, 34–37]. In the population-based Rotterdam scan study [15] there was a strong association between strictly lobar (but not deep) CMBs and APOE ε4 (later confirmed in a meta-analysis [38]), consistent with the well-known relation of this allele with CAA [1]. The APOE ε2 allele is also of major importance in CAA-related cerebral hemorrhage [39], although its exact role in CMBs mechanisms is still unexplored in the literature. In another healthy elderly cohort, multiple lobar CMBs were recently found to be associated with higher amyloid-β burden, as detected by 11C-Pittsburgh Compound B positron emission tomography (PiB-PET) imaging [40]. Co-registered PiB-PET imaging has shown that lobar CMB hotspots preferentially correspond to locations with increased amyloid concentration [41]. However, PiB-PET has poor spatial resolution and, similar to the other clinical-radiological studies on the topic, cannot reliably resolve the location of parenchymal and vascular amyloid-β. Thus, histopathological validation and studies on the pathogenesis of CMB at the level of individual vessel pathology are crucial to supplement imaging studies.

One of our main results is the spatial dissociation between CMB and CAA that was observed on close neuropathological examination. CAA predominantly affected pial arteries, and their superficial cortical branches; in contrast, CMB were found at the level of white matter cortical perforating vessels or medullary arteries. This is consistent with a study of 8 AD patients by Schrag and colleagues [12] who reported that the majority of pathologically-defined CMB occurred near the cortical ribbon in the adjacent white matter. In a larger autopsy series, Tanskanen et al. [6] mentioned that even in the simultaneous presence of CMB and CAA on the same histological slide, CMBs were not always situated around amyloid-laden vessels. Fisher et al. [32] in their series of 33 cases also report the occurrence of CMB around vessels without any evidence of amyloid deposition. The neuropathological studies that report a direct relationship between CAA and CMB are scarce and based on a very small number of subjects [12, 21]. In a series of 6 autopsy cases with CAA that included 3 cases with high CMB counts, 2 cases with a low number of CMB and 1 case without any microbleed, Greenberg and collaborators observed a thicker amyloid positive vessel wall in subjects with numerous microbleeds [21]. Schrag et al. [12] performed postmortem MRI in 8 AD autopsy cases and found advanced CAA in 6 of them, with amyloid deposion in the vessel’s wall near microbleeds.

Our study has several limitations which should taken into account. It was peformed in a hospital based autopsy series with a high mean age, and may represent a more severely affected population than community based samples, where the frequency of CMB might be lower. Also, we report on the presence or absence of CMB,including very small lesions, clearly identifiable on neuropathological examination but probably very hard to visualize on conventional MRI. Finally, we have analyzed the relation between lobar CMB and CAA pathology, regardless of the concurrent presence in the same brain of CMB in deep gray matter or brainstem CMB, which might indicate the presence and contribution of non-CAA small vessels disease pathology in the pathophysiology of microbleeds.

## Conclusions

Our study does not intend to underemphasise the clinical importance of the *in vivo* detection of MRI-defined CMB or critically analyze the current MRI Boston diagnostic criteria, since it does not contain either MRI or clinical data. However, our neuropathological data provide evidence that the assumed link between CAA and lobar CMB, an MRI derived concept, does not seem to have a clear direct neuropathological validity, at least at the level of individual vessel pathology. Of note, there seems to be a dissconnection of the MRI-definition vs. pathological definition of CMBs. For example, CMBs detected on 3 T blood-sensitive sequences are likely mostly very much larger than the few hemosiderin deposits that are observed microscopically. The literature on CMBs overwhelmingly emphasizes them as a haemorrhagic signature and stress the hemorrhagic risk associated with microbleeds. However, some authors have postulated that parenchymal iron deposits may arise both from haemorrhagic and ischaemic mechanisms. Therefore the field is using the same terminology to describe what may be different types of lesion - a more definitive radiographic imaging marker, with as-yet uncertain pathologic basis [7]. Further studies will need to explore additional potential pathophysiological mechanisms that could lead to the occurrence of CMB, including direct imaging-pathologic correlation studies and ex-vivo MRI data.
